# The Genus *Pachyma* (Syn. *Wolfiporia*) Reinstated and Species Clarification of the Cultivated Medicinal Mushroom “Fuling” in China

**DOI:** 10.3389/fmicb.2020.590788

**Published:** 2020-12-15

**Authors:** Fang Wu, Shou-Jian Li, Cai-Hong Dong, Yu-Cheng Dai, Viktor Papp

**Affiliations:** ^1^Institute of Microbilogy, School of Ecology and Nature Conservation, Beijing Forestry University, Beijing, China; ^2^State Key Laboratory of Mycology, Institute of Microbiology, Chinese Academy of Sciences, Beijing, China; ^3^Beijing Advanced Innovation Center for Tree Breeding by Molecular Design, Beijing Forestry University, Beijing, China; ^4^Institute of Horticultural Plant Biology, Szent István University, Budapest, Hungary

**Keywords:** *Poria cocos*, *Daedalea extensa*, *Macrohyporia*, Hoelen, phylogeny, nomenclature

## Abstract

The fungus “Fuling” has been used in Chinese traditional medicine for more than 2000 years, and its sclerotia have a wide range of biological activities including antitumour, immunomodulation, anti-inflammation, antioxidation, anti-aging etc. This prized medicinal mushroom also known as “Hoelen” is resurrected from a piece of pre-Linnean scientific literature. Fries treated it as *Pachyma hoelen* Fr. and mentioned that it was cultivated on pine trees in China. However, this name had been almost forgotten, and *Poria cocos* (syn. *Wolfiporia cocos*), originally described from North America, and known as “Tuckahoe” has been applied to “Fuling” in most publications. Although Merrill mentioned a 100 years ago that Asian *Pachyma hoelen* and North American *P. cocos* are similar but different, no comprehensive taxonomical studies have been carried out on the East Asian *Pachyma hoelen* and its related species. Based on phylogenetic analyses and morphological examination on both the sclerotia and the basidiocarps which are very seldomly developed, the East Asian samples of *Pachyma hoelen* including sclerotia, commercial strains for cultivation and fruiting bodies, nested in a strongly supported, homogeneous lineage which clearly separated from the lineages of North American *Wolfiporia cocos* and other species. So we confirm that the widely cultivated “Fuling” *Pachyma hoelen* in East Asia is not conspecific with the North American *Wolfiporia cocos*. Based on the changes in Art. 59 of the International Code of Nomenclature for algae, fungi, and plants, the generic name *Pachyma*, which was sanctioned by Fries, has nomenclatural priority (ICN, Art. F.3.1), and this name well represents the economically important stage of the generic type. So we propose to use *Pachyma* rather than *Wolfiporia*, and subsequently *Pachyma hoelen* and *Pachyma cocos* are the valid names for “Fuling” in East Asia and “Tuckahoe” in North America, respectively. In addition, a new combination, *Pachyma pseudococos*, is proposed. Furthermore, it seems that *Pachyma cocos* is a species complex, and that three species exist in North America.

## Introduction

In the subkingdom Dikarya, many fungi can produce dense aggregations called sclerotia to survive challenging environmental conditions and to provide reserves for fungi to germinate (Coley-Smith and Cooke, 1971; Willets and Bullock, 1992; Smith et al., 2015). Sclerotia, as persistent fungal structures commonly contain biologically active secondary metabolites, are used as a functional food (Wong and Cheung, 2009; Lau and Abdullah, 2016). Large subterranean sclerotia of different mushroom species are traditionally consumed by indigenous people around the world (Oso, 1977; Aguiar and Sousa, 1981; Bandara et al., 2015; Lau et al., 2015). In North America, the hypogeous sclerotia of a mushroom species, know as “Tuckahoe” or “Indian bread,” are utilized as a traditional food by native Americans (Gore, 1881; Weber, 1929). The first valid scientific description of this fungal sclerotia was given by Schweinitz (1822), who named it *Sclerotium cocos* Schwein. This name was accepted by Fries (1822), when he proposed the genus *Pachyma* Fr. Subsequently, the name *Pachyma cocos* (Schwein.) Fr. became the most popular binomial of the Tuckahoe mushroom (e.g., Currey and Hanbury, 1860; Gore, 1881; Prilleaux, 1889; Elliott, 1922). However, the sexual stage of *P. cocos* had remained unknown for a 100 years, until its whitish resupinate poroid fruiting body was discovered by Wolf (1922). At that time, the generic name *Poria* Pers. was widely used for all light-colored and resupinate polypores (Murrill, 1920, 1923), thus the sexual stage was named as *Poria cocos* (Schwein.) F. A. Wolf by Wolf (1922). The classification of *Poria cocos* was revised by Johansen and Ryvarden (1979), who transferred this species to their new genus *Macrohyporia* I. Johans. & Ryvarden, typified by *M*. *dictyopora* (Cooke) I. Johans. & Ryvarden. Later, Ryvarden and Gilbertson (1984) established the genus *Wolfiporia* Ryvarden & Gilb. typified by *Poria cocos*, based on its different spore morphology similar to *M*. *dictyopora*. However, Ginns and Lowe (1983) placed *Poria cocos* in synonymy with the earlier teleomorphic name *Daedalea extensa* Peck, and transferred this species to *Macrohyporia*. Subsequently, Ginns (1984) accepted the generic revision of Ryvarden and Gilbertson (1984) and corrected the name of the species by publishing the binomial, *Wolfiporia extensa* (Peck) Ginns. Nevertheless, because of the research and the preference of the traditional medical community to continue using the familiar name “cocos,” Redhead and Ginns (2006) proposed to conserve the name *Poria cocos* (syn. *Wolfiporia cocos*) over *Daedalea extensa* (syn. *Wolfiporia extensa*). Finally, the conservation of *Poria cocos* was recommended by the Nomenclature Committee for Fungi (Norvell, 2008).

The name *Poria cocos* is also commonly applied to a fungal sclerotium, known as “Fuling” in China, which has been used in Chinese traditional medicine for more than 2000 years for relieving coughs, inducing diuresis, aleviating anxiety, relieving fever, antitumor; adjustment of intestinal bacterial flora, antihyperlipidemic activity, antioxidant, anti-hepatitis B virus, anti-inflammation, anti-metastasis, anti-tyrosinase, hypoglycemic activity, improvement of cardiac function, improvement of learning and memory abilities, improvement of liver fibrosis, prevention of diabetic nephropathy, and sedative and hypnotic activities (Wang et al., 2013; Wu et al., 2019). Pharmacological studies have confirmed these properties (e.g., Sun, 2014; Zhang et al., 2018; Li et al., 2019). The edible sclerotia of “Fuling” is widely cultivated in China (Wang et al., 2013), and the products are exported to more than 40 countries (Chi et al., 2018). This prized medicinal mushroom is also known as “Hoelen” (e.g., Xu et al., 2014; Li et al., 2016; Sun et al., 2016), a name erected from a piece of pre-Linnean scientific literature published posthumously by Rumphius (1750). Fries (1822) mentioned “Hoelen” as a little-known medicinal species from China, under the genus *Pachyma*. Merrill (1917) noted that *Pachyma hoelen* Fr. was cultivated on pine trees in various parts of China and that it had been referred to as *Poria cocos*. In order to clarify the identity of *P*. *hoelen*, he sent a Chinese specimen (received from a drug store) for examination to W. A. Murrill. Murrill stated that the Chinese sclerotia showed similarity to the samples collected from different localities in America, but he thought “*that Pachyma hoelen Fries is distinct from P. cocos Fries*” (Merrill, 1917).

In recent years, molecular studies have shown that several traditionally used and widely cultivated East Asian medicinal mushrooms (e.g., *Auricularia heimuer* F. Wu, B.K. Cui & Y.C. Dai, *Flammulina filiformis* (Z.W. Ge, X.B. Liu & Zhu L. Yang) P.M. Wang, Y.C. Dai, E. Horak & Zhu L. Yang, *Ganoderma lingzhi* Sheng H. Wu, Y. Cao & Y.C. Dai) are different at the species level from their European or North American relatives (Cao et al., 2012; Wu et al., 2014; Dai et al., 2017; Wang et al., 2018). Currently, “Fuling” is widely identified with *Poria cocos* (syn. *Wolfiporia cocos*, syn. *Pachyma cocos*), a species originally described from North America, and no comprehensive taxonomical studies have been carried out on the East Asian *Pachyma hoelen* since it was described by Fries almost 200 years ago. Therefore, in this study we aim to typify the forgotten species *P. hoelen* and clarify the taxonomy of the “Fuling” mushroom, based on morphological features and phylogenetic evidence.

## Materials and Methods

### Morphological Studies

Specimens and isolates of *Pachyma* “*cocos*” originating from East Asia (China, Japan) and North America were examined, including wild collections and commercially cultivated strains. Voucher specimens are deposited at the herbarium of the Institute of Microbiology, Beijing Forestry University (BJFC) and Herbarium Mycologium, Chinese Academy of Sciences, Beijing, China (HMAS). The designated neotype of *Pachyma hoelen* (Dong 897, HMAS 248370) is registered in MycoBank (Robert et al., 2013). Macro-morphological descriptions are based on field notes and dry herbarium specimens. Microscopic measurements and drawings were made from slide preparations of dried specimens stained with Cotton Blue and Melzer's reagent following Dai (2010). In presenting spore size variation, 5% of measurements were excluded from each end of the range and this value is given in parentheses. The following abbreviations were used: KOH = 2% potassium hydroxide, CB– = acyanophilous, IKI– = neither amyloid nor dextrinoid in Melzer's reagent, L = mean spore length (arithmetic average of all spores), W = mean spore width (arithmetic average of all spores), Q = variation in the L/W ratios between specimens studied, n (a/b) = number of spores (a) measured from given number of specimens (b).

### Molecular Phylogenetic Study

Total genomic DNA was extracted from dried specimens using a CTAB rapid plant genome extraction kit (Aidlab Biotechnologies Company, Limited, Beijing, China) according to the manufacturer's instructions. To generate PCR amplicons, the following primer pairs were used: ITS4 and ITS5 (White et al., 1990) for the internal transcribed spacer (ITS), and 983F and 1567R (Rehner and Buckley, 2005) for a region of the translation elongation factor alpha-1 (*tef1*), LR0R and LR7 (Vilgalys and Hester, 1990) for the 28S gene region (LSU) and bRPB2-6F and bRPB2-7.1R (Matheny, 2005) for partial RNA polymerase II, second largest submit (*rpb2*). The PCR procedures followed Song and Cui (2017). PCR products were purified and sequenced at the Beijing Genomics Institute with the same primers. The sequences generated during this study are deposited in NCBI GenBank under the accession numbers MW251858-MW251879 (ITS and LSU), MW250253-MW250273 (*tef1* and *rpb2*) and listed in Table 1.

**Table 1:** Taxa used in the phylogenetic analyses along with their GenBank accession numbers and references.

**Species name**	**Collection number**	**Origin**	**ITS**	**LSU**	***tef1***	***rpb2***	**References**
*Antrodia serpens*	Dai 7465	China	KR605813	KR605752	KR610742	KR610832	Han et al., 2016
*Antrodia serpens*	Rivoire 3576 (LY)	France	KC543169	–	KC543191	–	Spirin et al., 2013
*Antrodia tanakae*	Kajander 270 (H)	Finland	KC543165	–	KC543190	–	Spirin et al., 2013
*Antrodia tanakae*	Spirin 3968 (H)	Russia	KC543164	–	KC543193	–	Spirin et al., 2013
*Antrodia heteromorpha*	Dai 12755	USA	KP715306	KP715322	KP715336	KR610828	Chen and Cui, 2015
*Antrodia heteromorpha*	Gaarder 1665 (O)	Norway	KC543150	–	KC543186	–	Spirin et al., 2013
*Antrodia heteromorpha*	CBS 200.91	Canada	DQ491415	–	–	DQ491388	Kim et al., 2007
*Fomitopsis betulina*	Dai 11449	China	KR605798	KR605737	KR610726	KR610816	Han et al., 2016
*Fomitopsis betulina*	Miettinen 12388	Finland	JX109856	JX109856	JX109913	JX109884	Binder et al., 2013
*Fomitopsis pinicola*	Cui 10312	China	KR605781	KR605720	KR610689	KR610780	Han et al., 2016
*Fomitopsis pinicola*	AT-Fp-1	Sweden	MK208852	–	MK236359	MK236362	Haight et al., 2019
*Fomitopsis schrenkii*	JEH-150, type	USA	KU169365	–	MK236356	MK208858	Haight et al., 2019
*Fomitopsis schrenkii*	JW24-525-0	USA	MK208854	–	MK236358	MK208860	Haight et al., 2019
*Fomitopsis durescens*	Overholts 4215	USA	KF937293	KF937295	–	–	Han, et al., 2014
*Fomitopsis durescens*	O 10796	Venezuela	KF937292	KF937294	KR610669	KR610766	Han et al., 2014
*Kusaghiporia usambarensis*	J. Hussein 01/16	Tanzania	–	MH010044	MH048871	MH048870	Hussein et al., 2018
*Kusaghiporia usambarensis*	J. Hussein 01/17	Tanzania	–	MH010045	MH048869	–	Hussein et al., 2018
*Laetiporus gilbertsonii*	JV 1109/31	USA	KF951293	KF951306	KX354630	KX354671	Song and Cui, 2017
*Laetiporus gilbertsonii*	CA 13	USA	EU402549	EU402527	AB472666	–	Lindner and Banik, 2008
*Laetiporus montanus*	Dai 15888	China	KX354466	KX354494	KX354619	KX354662	Song and Cui, 2017
*Laetiporus montanus*	L17-LI	Austria	EU840553	–	–	–	Vasaitis et al., 2009
*Laetiporus sulphureus*	JV 1106/15	Czech Republic	KF951296	KF951303	KX354609	KX354654	Song and Cui, 2017
*Laetiporus sulphureus*	Cui 12388	China	KR187105	KX354486	KX354607	KX354652	Song and Cui, 2017
*Pachyma cocos*	CBS 279.55	USA	**MW251869**	**MW251858**	**MW250253**	**MW250264**	**This study**
Pachmya cocos^*^	MD-106	USA	EU402594	EU402594	–	–	Lindner and Banik, 2008
Pachmya cocos^**^	JV0506_4J	USA	MN392911	MN392911	–	–	unpublished
Pachmya cocos^**^	JV1608_23J	USA	MN392912	MN392912	–	–	unpublished
Pachmya cocos^*^	CFMR:MD-275	USA	KU668964	–	–	–	unpublished
Pachmya cocos^*^	Batch3_14064_14098	USA	KT693239	–	–	–	Raja et al., 2017
Pachmya cocos^***^	MRM011	USA	MT241733	–	–	–	unpublished
*Pachmya hoelen*	CGMCC 5.908	China	**MW251870**	**MW251859**	**MW250254**	**MW250265**	**This study**
*Pachmya hoelen*	Dai 20041	China	**MW251878**	**MW251867**	**MW250262**	**MW250273**	**This study**
*Pachmya hoelen*	Dai 20036	China	**MW251877**	**MW251866**	**MW250261**	**MW250272**	**This study**
*Pachmya hoelen*	Dai 20034	China	**MW251879**	**MW251868**	**MW250263**	–	**This study**
*Pachmya hoelen*	Dong 750	China	**MW251873**	**MW251862**	**MW250257**	**MW250268**	**This study**
*Pachmya hoelen*	Dong 830	China	**MW251874**	**MW251863**	**MW250258**	**MW250269**	**This study**
*Pachmya hoelen*	Dong 829	China	**MW251875**	**MW251864**	**MW250259**	**MW250270**	**This study**
*Pachmya hoelen*	Dong 897	China	**MW251871**	**MW251860**	**MW250255**	**MW250266**	**This study**
*Pachmya hoelen*	Dong 906	China	**MW251872**	**MW251861**	**MW250256**	**MW250267**	**This study**
*Pachmya hoelen*	KCTC6480	Japan	**MW251876**	**MW251865**	**MW250260**	**MW250271**	**This study**
Pachmya hoelen^*^	XJ-28	China	KX268225	–	–	–	unpublished
Pachmya hoelen^*^	Taikong	China	KX268226	–	–	–	unpublished
Pachmya hoelen^*^	CBK-1	China	KX354453	KX354689	KX354688	KX354685	Song and Cui, 2017
*Pachyma pseudococos*	Dai 15269, type	China	KX354451	–	–	–	Tibpromma et al., 2017
*Phaeolus schweinitzii*	AFTOL-ID 702	USA	–	AY629319	DQ028602	DQ408119	Matheny et al., 2007
*Phaeolus schweinitzii*	OKM-4435-T	USA	–	KC585199	–	–	Ortiz-Santana et al., 2013
*Rhodofomes cajanderi*	Cui 9879	China	KC507157	KC507167	KR610663	KR610763	Han et al., 2016
*Rhodofomes cajanderi*	JV 0410/14a,b-J	USA	KR605768	KR605707	KR610664	–	Han et al., 2016
*Rhodofomes rosea*	JV 1110/9	Czech Republic	KR605783	KR605722	KR610694	KR610785	Han et al., 2016
*Rhodofomes rosea*	Cui 10633	China	KR605782	KR605721	KR610693	KR610784	Han et al., 2016
*Wolfiporia cartilaginea*	Dai 3764	China	KX354456	–	–	–	unpublished
*Wolfiporia cartilaginea*	13122	Japan	KC585405	–	–	–	Ortiz-Santana et al., 2013
*Wolfiporia cartilaginea*	O 913120	Japan	KX354455	–	–	–	unpublished
*Wolfiporia dilatohypha*	S.D. Russell MycoMap 7010	USA	MK564607	–	–	–	unpublished
*Wolfiporia dilatohypha*	FP94089	USA	EU402554	EU402518	–	–	Lindner and Banik, 2008
*Wolfiporia dilatohypha*	CS-63	USA	KC585400	EU402516	–	–	Lindner and Banik, 2008
*Wolfiporia dilatohypha*	FP-94089-R	USA	KC585401	KC585236	–	–	Ortiz-Santana et al., 2013
*Wolfiporia dilatohypha*	CS-63-59-13-A-R	USA	KC585400	KC585234	–	–	Ortiz-Santana et al., 2013
*Trametes suaveolens*	Cui 11586	China	KR605823	KR605766	KR610759	KR610848	Han et al., 2016
*Polyporus tuberaster*	Dai 11271	China	KU189769	KU189800	KU189914	KU189983	Zhou et al., 2016

* *as Wolfiporia cocos*;

** * as Macrohyporia cocos*;

*** *as Wolfiporia aff. extensa*.

*Sequences produced in this study are indicated in bold*.

Two datasets were used in the phylogenetic analyses. The multigene dataset was used to gain information about the phylogenetic position of the genus. The second ITS dataset represented sequences of only *Wolfiporia cocos*-related specimens. In the multigene phylogenetic analyses, the highly divergent ITS regions of the *Wolfiporia* s. str. (syn. *Pachyma*) specimens were removed. Sequences were aligned with the online version of MAFFT v. 7 using the E-INS-i algorithm (Katoh and Standley, 2013), under default settings. Each alignment was checked separately and edited with SeaView 4 (Gouy et al., 2010). Subsequently, the concatenated ITS + LSU + *tef1* + *rpb2* dataset alignment was subjected to Maximum Likelihood (ML) and Bayesian Inference (BI) phylogenetic analyses, which were performed in RaxmlGUI (Silvestro and Michalak, 2012) and MrBayes 3.1.2 (Ronquist and Huelsenbeck, 2003), respectively. ML analysis was done using 1,000 rapid ML bootstrap searches. Four partitions (ITS, LSU, *tef1, rpb2*) were set and the GTRGAMMA nucleotide substitution model was selected for each partition. Rapid bootstrap analysis with 1,000 replicates was applied for testing branch support. BI was performed with the GTR + Γ model of evolution. The same partition scheme was used as for the ML analysis (see above). The BI settings were: four Markov chain Monte Carlo (MCMC) over 5 million generations, sampling every 1000th generation, two independent runs, and burn-in of 20% (the first 1,000 trees were discarded). Post burn-in trees were used to compute a 50% majority rule consensus phylogram. Phylogenetic trees from both ML and BI analyses resulted in largely congruent topologies. The best scoring ML tree from the RAxML analysis was edited with MEGA6 (Tamura et al., 2013). ML bootstrap values (BS) > 70% and Bayesian posterior probabilities (PP) > 0.9 were considered evidence for statistical branch support.

## Results

### Molecular Phylogeny

**Figure 1:**
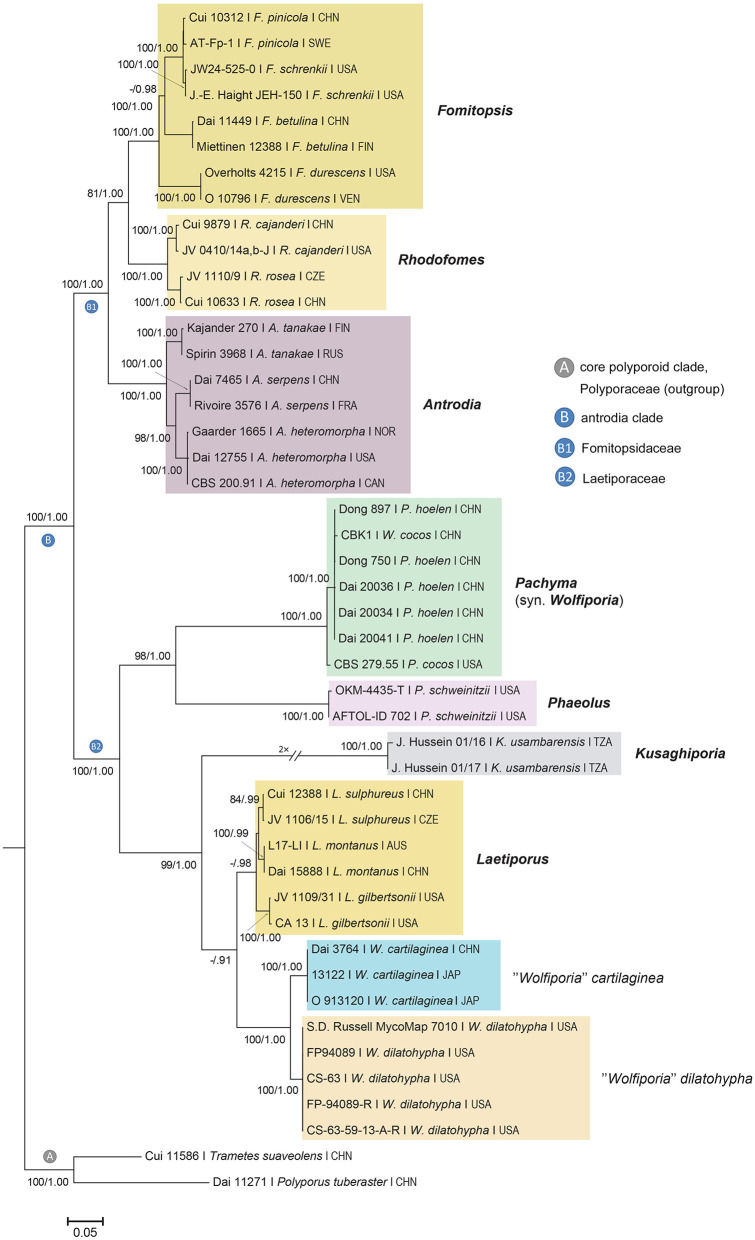
Phylogeny of the genus *Pachyma* (syn. *Wolfiporia*) within the antrodia clade inferred from RAxML and MrBayes analyses of the combined ITS–LSU–*tef1*–*rpb2* sequences. Topology is from the best scoring Maximum Likelihood (ML) tree. *Polyporus tuberaster* and *Trametes suaveolens* served as the outgroup. Bayesian Posterior Probabilities (BPP) > 0.9 and ML bootstrap values > 70% are shown above or below branches. The bar indicates 0.05 expected change per site per branch.

**Figure 2:**
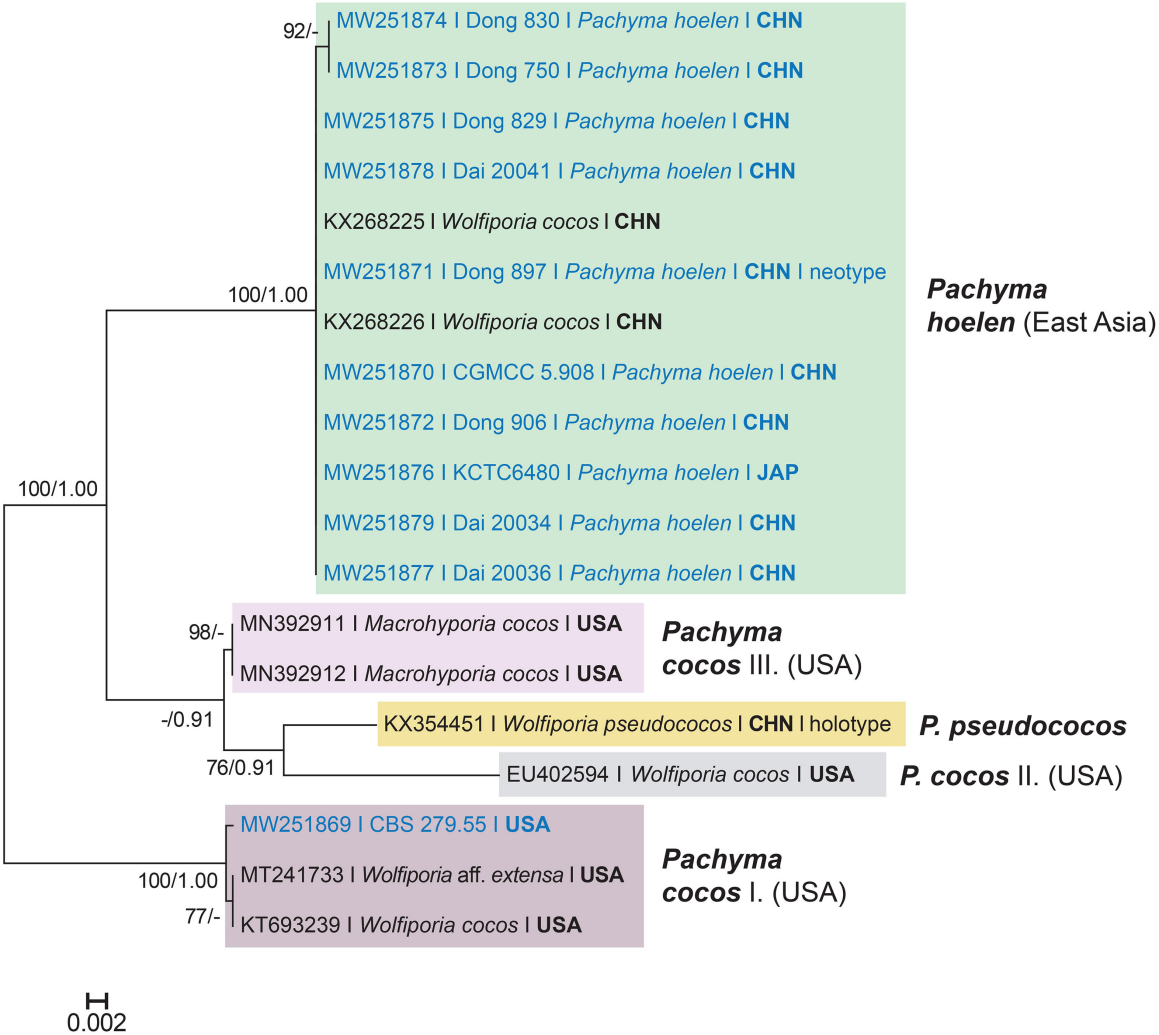
Phylogeny of *Pachyma hoelen* and related taxa inferred from RAxML and MrBayes analyses of nrDNA ITS sequences. Topology is from the best scoring Maximum Likelihood (ML) tree. Bayesian Posterior Probabilities (BPP) > 0.9 and ML bootstrap values > 70% are shown above or below branches. The bar indicates 0.002 expected change per site per branch.

The multigene and ITS phylogenetic analyses were carried out using two datasets comprising 46 taxa and 3,160 characters, and 19 taxa and 1,698 characters including gaps, which were treated as missing data. The phylogenetic tree topology of the concatenated ITS-LSU-*tef1*-*rpb2* dataset (Figure 1) is largely congruent with previously published phylogenies (e.g., Ortiz-Santana et al., 2013; Justo et al., 2017; Hussein et al., 2018) and the genus *Pachyma* (syn. *Wolfiporia*) clustered in the Laetiporaceae Jülich (syn. Phaeolaceae Jülich) within the antrodia clade. At the species level, the neotype of *Pachyma hoelen* (Dong 897, HMAS 248370) and other studied specimens from East Asia (incl. Dong 750, which is the widely cultivated strain now in China) represent a well-supported (ML/BA 100/1.00), relatively homogeneous clade. Analysis of ITS sequences (Figure 2) also shows that all newly sequenced strains from East Asia are nested in a strongly supported clade (ML/BA 100/1.00). This clade is clearly separated from the other clades in the phylogeny where *P. cocos* strains from North America and the holotype of *W*. *pseudococos* (GenBank no. KX354451) are nested (Figure 2). In the ITS phylogenetic tree, the *Wolfiporia cocos* and *Macrohyporia cocos* samples from the United States separated into three distinct clades and they are not closely related to *Pachyma hoelen* in phylogeny. Our phylogenetic reconstruction of the ITS sequences indicates that the North American samples identified as *W*. *cocos* and deposited in GenBank cover more than one species. The newly sequenced *P*. *cocos* isolate (CBS 279.55), originating from South Carolina (Southeastern United States), forms a well-supported (ML/BA 100/1.00) lineage with two sequences originating from the United States (GenBank no. MT241733 and KT693239). The *W*. *cocos* specimen collected from hardwood species (*Alnus*) from the United States (Lindner and Banik, 2008) formed a separate lineage within a moderately supported clade (ML/BA 63/0.91) and grouped with the type of *W*. *pseudococos* and two unpublished sequences of *Macrohyporia cocos* (GenBank no. MN392911 and MN392912). Based on the above single-locus and multigene molecular data, the forgotten East Asian species, *Pachyma hoelen*, which is widely cultivated in China and Japan, is not conspecific with the North American *P*. *cocos* (syn. *Wolfiporia cocos*).

## Taxonomy

***Pachyma*** Fr., Syst. mycol. 2(1): 242 (1822)

Synonyms. *Gemmularia* Raf. per Steud., Nomencl. bot. P1. crypt.: 183 (1824); *Tucahus* Raf., Anal. Nat. Tabl. Univ. 2: 270 (1830) nom. illegit. (ICN; Art. 52.); *Rugosaria* Raf., Anal. Nat. Tabl. Univ. 1: 181 (1833) nom. illegit. (ICN; Art. 52.).

*Wolfiporia* Ryvarden and Gilb., Mycotaxon 19: 141 (1984)

*Generic type species: Pachyma cocos* (Schwein.) Fr., Syst. mycol. 2(1) 242 (1822) (Basionym. *Sclerotium cocos* Schwein., Schr. naturf. Ges. Leipzig 1: 56. 1822), selected by Donk (1962: 94).

*Description*. *Sclerotia* globose or irregularly shaped, when fresh, outer crust reddish brown, inner context white and corky; outer crust becomes hard corky and inner context becomes fragile when dry. *Basidiocarp* annual, resupinate; *pore surface* cream to ash gray when fresh; *hyphal system* dimitic, generative hyphae with simple septa, skeletal hyphae thick-walled, distinctly thicker than generative hypha; *cystidia* absent, but cystidioles occasionally present; *basidia* clavate, with four sterigmata and a simple basal septum; *basidiospores* cylindrical, ellipsoid, hyaline, thin-walled, IKI–, CB–. *Rot type* brown.

*Nomenclatural remarks*. Fries (1822) described the anamorphic genus *Pachyma* and distinguished three species. Later, Donk (1962) designeted the first species, *P*. *cocos* (Schwein.) Fr. (syn. *Sclerotium cocos* Schwein.) as the generic type of *Pachyma*. The teleomorphic genus *Wolfiporia* was typified with *Poria cocos* F. A. Wolf by Ryvarden and Gilbertson (1984), which was a species derived from *Sclerotium cocos* Schwein., hence it was cited as a basionym by Wolf (1922). Therefore, both *Pachyma* and *Wolfiporia* are typified with *Sclerotium cocos* Schwein., thus these genera are considered as synonyms. Based on the changes in Art. 59 of the International Code of Nomenclature for algae, fungi, and plants (ICN; Turland et al., 2018), all legitimate fungal names are treated equally for the purposes of establishing priority, regardless of the life history stage of the type (Art. F.8.1). In the case that the sexually typified generic name does not have priority it is recommended that it can either be formally conserved (e.g., Braun, 2013), or included on a list of protected names (Rossman, 2014). The generic names *Pachyma* and *Wolfiporia* are both listed by Kirk et al. (2013) for protection as a result of changes in Art. 59. However, the earlier name *Pachyma* is sanctioned by Fries (ICN, Art. F.3.1) and well represents the economically important stage of the generic type. For this reason, currently we consider that it is unnecessary to conserve the name *Wolfiporia* over *Pachyma*. Consequently, based on nomenclatural priority, the use of the earlier and sanctioned generic name *Pachyma* is recommended over *Wolfiporia*.

**Figure 3:**
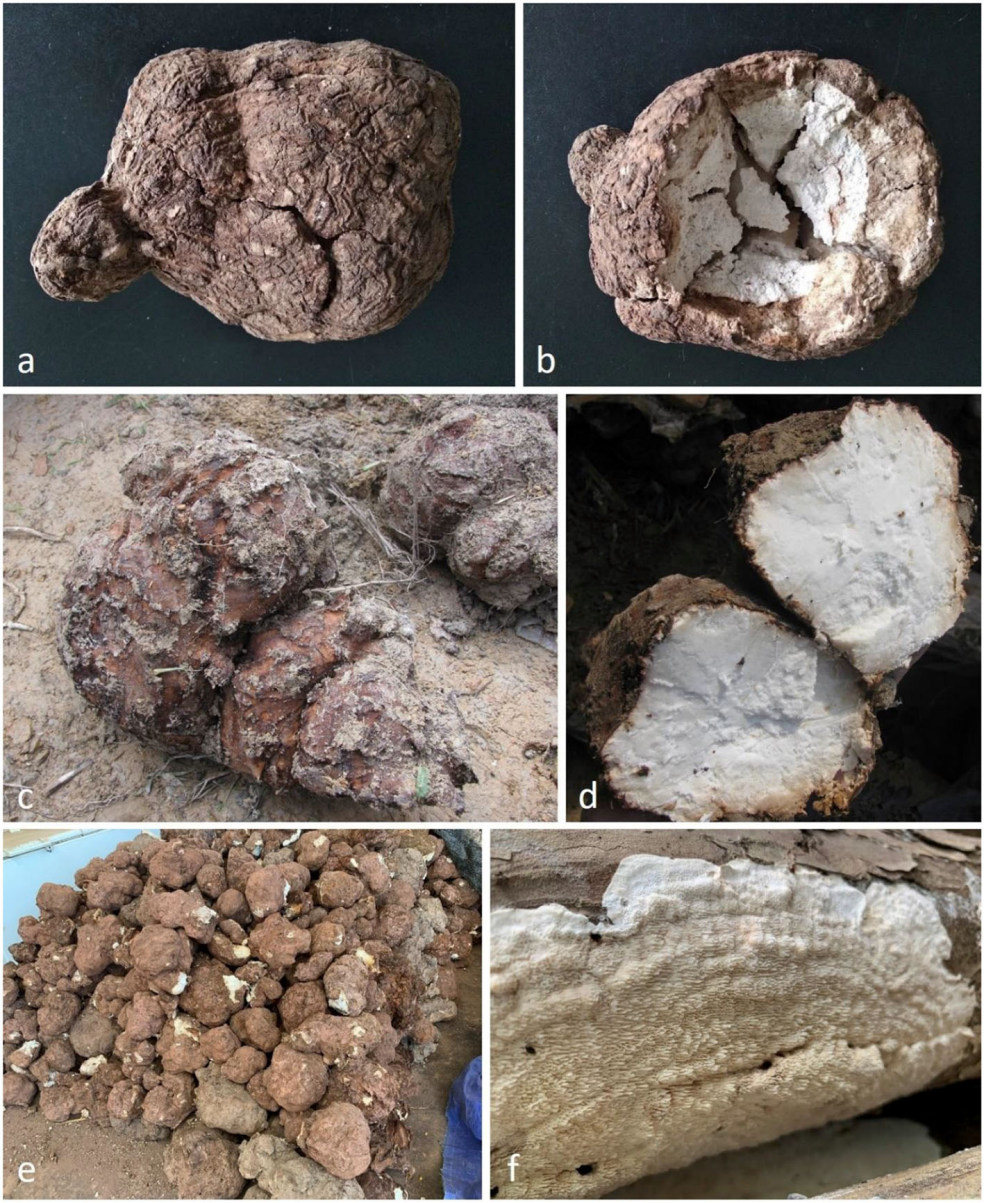
Sclerotia and basidiome of *Pachyma hoelen*. **(a,b)** Dry sclerotium of *P. hoelen* (neotype Dong 897, HMAS 248370). **(c–e)** Fresh sclerotia of *P. hoelen*. f. Basidiome of *P. hoelen* (Dai 20036). Photos (**a,b**: SJ. Li, **c–f**: Y.C. Dai).

**Figure 4:**
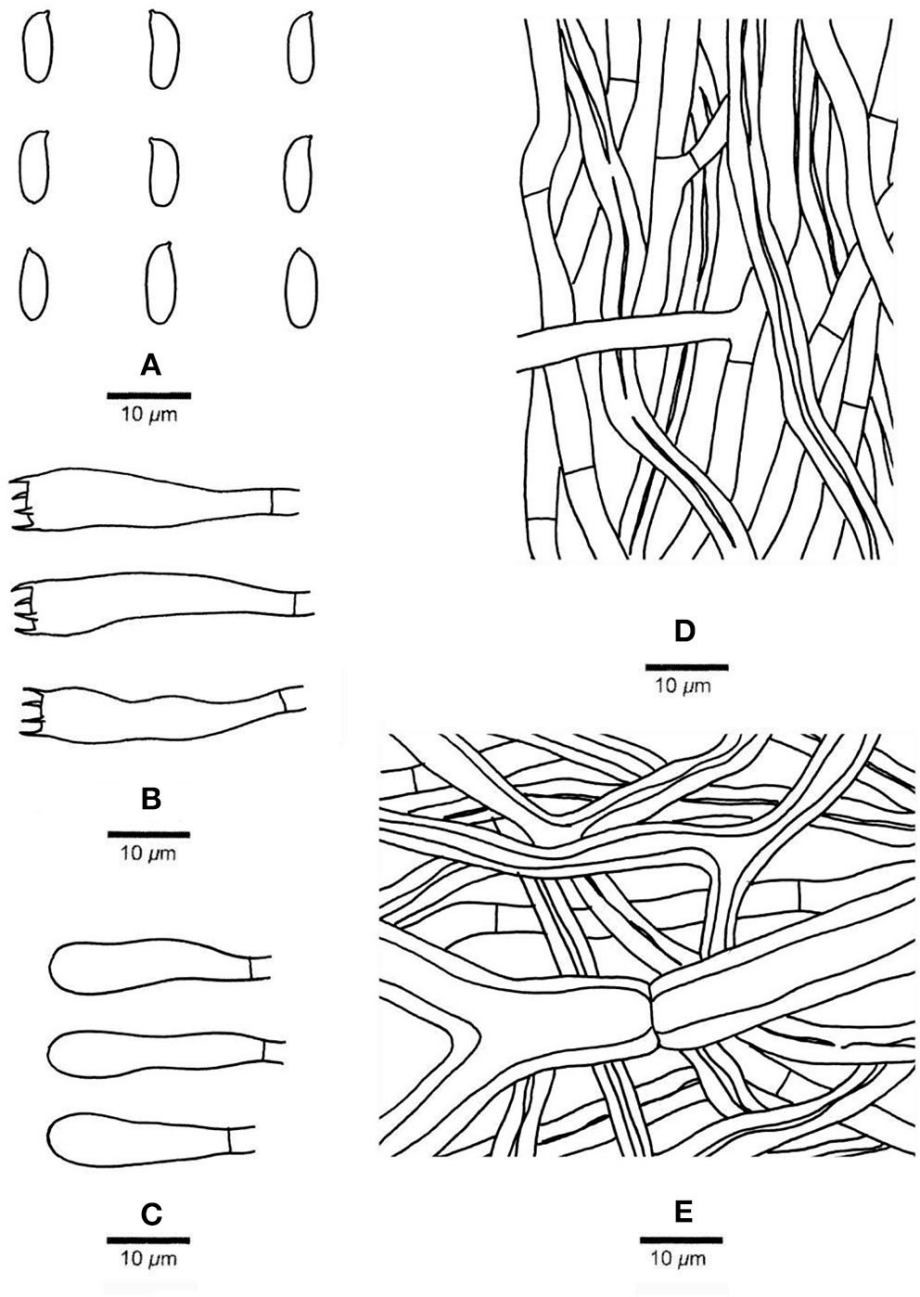
Microscopic structures of *Pachyma hoelen* basidiome (Dai 20036). **(A)** Basidiospores. **(B)** Basidia. **(C)** Basidioles. **(D)** Hyphae from trama. **(E)** Hyphae from subiculum. Drawing by S. L. Liu.

***Pachyma hoelen*** Fr., Syst. mycol. (Lundae) 2(1): 243 (1822) (Figures 3, 4)

*Description*. *Sclerotia* globose, subglobose, oval to irregularly shaped, up to 28 cm long and 22 cm wide, weighing up to 20 kg; when fresh, outer crust reddish brown, inner context white and corky; outer crust becomes hard corky and inner context becomes fragile when dry. *Basidiocarp* annual, resupinate, soft corky and without odor or taste when fresh, hard corky to fragile when dry, up to 20 cm long, 10 cm wide, 5.5 mm thick at center. *Margin* thin, usually pores extend to the very edge. *Pore surface* cream to ash gray when fresh, becoming pinkish buff to cinnamon buff when dry, not glancing; pores round, angular or sinuous, 1–2 per mm; dissepiments thick, slightly lacerate to distinctly dentate. *Subiculum* cinnamon buff, hard corky, up to 1.5 mm; *tubes* hard corky to fragile, buff, up to 4 mm long. *Hyphal system* dimitic in all parts, generative hyphae with simple septa, skeletal hyphae dominant, all hyphae IKI–, CB–, weakly inflated in KOH. *Subicular hyphal structure* homogeneous, hyphae strongly interwoven; generative hyphae occasionally present, hyaline, thin-walled, occasionally branched, frequently simple septate, 4–6 μm in diam.; *skeletal hyphae* dominant, hyaline, thick-walled with a distinct wide lumen, usually flexuous, frequently branched, occasionally simple septate, 6–12 μm in diam. *Tramal generative hyphae* frequent, hyaline, thin-walled, occasionally branched, frequently simple septate, 3–5 μm in diam.; *tramal skeletal hyphae* frequent, hyaline, thick-walled with a wide lumen, flexuous, occasionally frequently branched and simple septate, 4–8 μm in diam. *Cystidia* and cystidioles absent; *basidia* clavate, with four sterigmata and a simple basal septum, 25–32 × 7–8 μm, basidioles in shape similar to basidia but slightly smaller. *Basidiospores* oblong-ellipsoid to cylindrical, tapering at apiculus, hyaline, thin-walled, IKI–, CB–, (6–)7–9.6(−11) × (2.5–)2.9–4(−4.1) μm, L = 8.24 μm, W = 3.2 μm, Q = 2.49–2.66 (*n* = 90/3). *Rot type* brown.

*Specimens examined*. China, Yunnan Province, Yongsheng County, Renhe, Yina, 21 Dec 2018, CH Dong 897 (HMAS 248370, neotype, designated here, MycoBank MBT394794); Guangxi Auto Region, Baise, Baise Park, on stump of *Pinus massoniana* 1 July 2019, Dai 20034 (BJFC031708), Dai 20036 (BJFC031710), Dai 20041 (BJFC031715).

*Nomenclatural remarks*. The name “Hoelen” is derived from Rumphius (1750), and frequently cited as *Pachyma hoelen* Rumph. in scientific literature (e.g., Saccardo et al., 1889; Hino and Katô, 1930; Takeda, 1934). In the work of Rumphius (1750) it is mentioned under the species *Tuber regium* Rumph. [nom. inval., Art. 32.1(a); current name is *Pleurotus tuber-regium* (Fr.) Singer], but without the name *Pachyma*, the genus which was introduced by Fries (1822). Although, Fries (1822) presumably adopted the description of *P*. *hoelen* from Rumphius (1750), this work was not cited by him. Therefore, the names *P*. *hoelen* Rumph. and *P*. *hoelen* Rumph. ex Fr. are incorrect interpretations. However, “Hoelen” formally was not clearly discussed by Fries (1822) as a binomial like the other two taxa, i.e., *P*. *cocos* (Schwein.) Fr. and *P*. *tuber-regium* Fr. (see also Donk, 1962). This nomenclatural uncertainty is supported by the index of the same work (Fries, 1822, p. 608), where *P*. *hoelen* was not listed under the genus *Pachyma* like the other two species. However, in his later work Fries (1832) clearly indicated that he accepted *P*. *hoelen* as a distinct species in the genus *Pachyma*. When Fries (1822) proposed the new genus *Pachyma*, he noted that “Hoelen” is a little-known species and marked it with a separate serial number (like the other two species) under the genus. Given that the epithet “Hoelen” can be assigned to the generic name *Pachyma*, and the species has a short diagnosis, the name *Pachyma hoelen* Fr. was published validly by Fries (1822) and sanctioned by the ICN (Art. F.3.1).

***Pachyma pseudococos*** (F. Wu, J. Song & Y.C. Dai) F. Wu, Y.C. Dai & V. Papp, **comb. nov**.

Basionym. *Wolfiporia pseudococos* F. Wu, J. Song & Y.C. Dai, Fungal Diversity 83: 237 (2017)

MycoBank MB838018.
*Description*. For the description, see Tibpromma et al. (2017)
*Specimen examined*. CHINA, Hainan Province, Ledong County, Jianfengling Nature Reserve, on dead angiosperm tree, 1 June 2015, Dai 15269 (BJFC019380, holotype).

Remarks. New combination is proposed for *Wolfiporia pseudococos* in *Pachyma* based on molecular data and morphological features of the basidiocarp. Ecologically, *P*. *pseudococos* grows on angiosperm trees in tropical China, while *P. hoelen* has a distribution in temperate areas and usually grows on conifers. Phylogenetically, the two species are closely related, but *P*. *pseudococos* forms a separate lineage based on the analyses of ITS sequences (Figure 2). The basidiocarps of *P. hoelen* shares similar morphological characteristics with *P. pseudococos*, but differs by the absence of cystidioles, and longer and thinner basidia (25–32 × 7–8 μm vs. 16–25 × 10–14 μm in *P. pseudococos*).

## Discussion

Before the introduction of the One Fungus-One Name (1F1N) concept, the correct name was the earliest legitimate name typified by the perfect state (= teleomorph). However, based on the changes in Art. 59 of the ICN (Turland et al., 2018), the legitimate generic names typified by anamorphic fungal stages are treated equally for the purposes of establishing priority. The generic names *Pachyma* and *Wolfiporia* have types that represent the same species and are thus synonyms. Since *Pachyma* is the earliest name and sanctioned by the ICN (Art. F.3.1), and has priority, we recommend *Pachyma* rather than *Wolfiporia*.

Based on molecular phylogenetic analysis, for the time being we accept two species in the genus *Pachyma* from China. We found that the tested wild specimens and commercial cultivars known as “Fuling” represent a single species and are not identical with the true *Pachyma cocos* (syn. *Poria cocos*) from North America. Based on a thorough study of the literature, the forgotten Friesian binomial *Pachyma hoelen* has proved to be the correct scientific name for this widely cultivated East Asian edible and medicinal mushroom. In addition, *Pachyma hoelen* grows in wood of *Pinus* exclusively in China, and it is cultivated on pine wood, too. *Pachyma cocos* sensu lato is widely distributed in North America, and both the sclerotia and basidiocarps grow on various angiosperm and gymnosperm hosts (Davidson and Campbell, 1954; Lowe, 1966). A few European locations of *P*. *cocos* have also been reported from France, Austria and Switzerland (Prilleaux, 1889; Bernicchia and Gorjón, 2020). Based on the high variability observed in the sequenced ITS region of *P*. *cocos* samples from the Americas, three taxa seems to be existed in North America, but no voucher teleomorphic samples of these taxa were studied, so we currently treat them as *Pachyma cocos* I*, Pachyma cocos* II and *Pachyma cocos* III. Further studies are needed to clarify the taxonomy of this species.

The other two validly described species formerly discussed in *Pachyma* are excluded from the genus, namely *P*. *tuber-regium* Fr. and *P*. *woermannii* J. Schröt. (Fries, 1822; Cohn and Schröter, 1891). The current name of the former species is *Pleurotus tuber-regium* (Fr.) Singer, a well-known edible and medicinal mushroom (Dai et al., 2009, 2010; Wu et al., 2019) native to the tropics, including Africa, Asia, and Australasia (Karunarathna et al., 2016). *Pachyma woermannii* presumably represents the same species and is identical with *Pleurotus tuber-regium*. The sclerotia (as *Pachyma woermannii*) and the lamellate basidiocarps (as *Lentinus woermannii* Cohn and J. Schröt.) of the same fungus were described at the same time by Cohn and Schröter (1891), based on specimens collected from Cameroon (Central Africa). Further study is needed to confirm if the both are interspecific.

The teleomorphic genus *Wolfiporia* contains eight legitimate names (Index Fungorum 2020), from which six species are accepted (He et al., 2019; Wijayawardene et al., 2020). However, amongst these, only two species (*Pachyma hoelen* and *P. pseudococos*) are confirmed in *Pachyma* by phylogenetic data so far (Figures 1, 2). Therefore, further phylogenetic and type studies are needed to clarify the systematic position of those *Wolfiporia* species, which are currently not accepted in *Pachyma*. *Wolfiporia castanopsis* Y.C. Dai was described by Dai et al. (2011) from Southwest China (Yunnan Province, Zixishan Nature Reserve), based on a specimen growing on the wood of *Castanopsis orthacantha* Franch. Morphologically, this species is closely related to *Pachyma cocos*, the type species of the genus *Pachyma*. The two species have similar poroid and resupinate basidiocarps, but *Wolfiporia castanopsis* has broadly ellipsoid basidiospores (7.6–10 × 5–7 μm, Dai et al., 2011). *Wolfiporia curvispora* Y. C. Dai was described from Northeast China (Jilin Province), based on a single collection growing on *Pinus koraiensis* Siebold & Zucc. Morphologically, *W*. *curvispora* differs from other species in *Wolfiporia* by its biennial habit, small pores (4–5 per mm), small, curved and cylindrical basidiospores (3.3–4.1 × 1.2–1.8 μm, Dai, 1998). *Wolfiporia cartilaginea* Ryvarden was described from Northeast China (Jilin Province, Changbaishan National Nature Reserve) (Ryvarden et al., 1986) and phylogenetically found to be closely related to *W. dilatohypha* Ryvarden & Gilb. (syn. *Poria inflata* Overh.); these two species formed a separate lineage that is closely related to, but distinct from the core *Laetiporus* clade (Banik et al., 2010; Hussein et al., 2018; Figure 1). *Wolfiporia sulphurea* (Burt) Ginns (syn. *Merulius sulphureus* Burt) has similar morphological characteristics to *Pachyma cocos*, but it causes a white rot (Ginns, 1968; Ginns and Lowe, 1983).

The primary fungal barcoding marker, ITS, is quite useful to separate most fungal species (Xu, 2016), but it is not enough for some groups if we only use ITS in their phylogeny (Lücking et al., 2020). Unusually, the ITS sequence of *Pachyma* is at least twice as long as the sequences for most taxa in the antrodia clade, which is presumably due to the insertions in the ITS1 and ITS2 regions (Lindner and Banik, 2008). Although, in general the thresholds ranging from 97.0 to 99.5% sequence similarity were the most optimal values for delimiting species in the Agaricomycetes (Blaalid et al., 2013; Garnica et al., 2016; Nilsson et al., 2019), Raja et al. (2017) believed that a larger threshold value (<97%) is acceptable in the case of *P*. *cocos* specimens, due to the presence of introns. The difference in the sequences of the neotype of *P*. *hoelen* compared with the *P*. *cocos* specimen from the USA (CBS 279.55) was 8.0% for ITS. In the comparison of *P*. *hoelen* and *P*. *cocos* secondary barcoding markers (incl. protein-coding genes) we found moderate, but significant differences between the two species: *tef1* (97.8%), *rpb2* (98%). Therefore, both nuclear ribosomal RNA genes (ITS) and protein-coding genes (*tef1, rpb2*) showed remarkable differences between *P*. *cocos* and *P*. *hoelen* with low intragroup heterogeneity in the later. This confirms the separation of the two species and suggests that the inclusion of additional markers (i.e., protein-coding genes) should be necessary for further studies on the genus *Pachmya*.

In conclusion, *Poria cocos* (syn. *Wolfiporia cocos*) has been applied to the prized Chinese medicinal mushroom “Fuling,” according to changes in Art. 59 of the International Code of Nomenclature for algae, fungi and plants, its correct binomial name is *Pachyma hoelen* Fr. which was validly published by Fries and sanctioned by the ICN. The wild teleomorphic stage (basidiocarps) of *Pachyma hoelen* is found and collected as the first time in China, and both tested wild specimens and commercial cultivars known as “Fuling” represent a single species. The illustrated description of *Pachyma hoelen* is given based on wild fruiting bodies and cultivated sclerotia, and its neotype is designated. The Chinese “Fuling” *Pachyma hoelen* is different from North American “Tuckahoe” *Pachyma cocos* (syn. *Wolfiporia cocos*), and *Pachyma* is recommended over *Wolfiporia* because it is the earliest and sanctioned generic name. Accordingly, *Pachyma cocos* (Schwein.) Fr. is the valid name for “Tuckahoe” in North America, and three taxa are existed among *Pachyma cocos* sensu lato. Currently five taxa are accepted in *Pachmya: P. hoelen, P. pseudococos, P. cocos* I*, P. cocos* II and *P. cocos* III. The phylogeny of other taxa previously described or combined in *Wolfiporia* are not analyzed, and their taxonomy is uncertain without molecular data.

## Data Availability Statement

The datasets presented in this study can be found in online repositories. The names of the repositories and accession numbers can be found in the article materials.

## Author Contributions

Y-CD and VP designed the experiments. FW, S-JL, and CH-D prepared the samples. VP conducted the molecular experiments and analyzed the data. FW, S-JL, CH-D, Y-CD, and VP revised the manuscript. All authors contributed to the article and approved the submitted version.

## Conflict of Interest

The authors declare that the research was conducted in the absence of any commercial or financial relationships that could be construed as a potential conflict of interest.
